# Editorial: Engineering microalgal chassis cells

**DOI:** 10.3389/fmicb.2023.1237999

**Published:** 2023-06-26

**Authors:** Xuefeng Lu, Martin Hagemann, Jin Liu, Pratyoosh Shukla, Xiaoming Tan

**Affiliations:** ^1^Qingdao Institute of Bioenergy and Bioprocess Technology, Chinese Academy of Sciences (CAS), Qingdao, China; ^2^Institute of Biosciences, Department of Plant Physiology, University of Rostock, Rostock, Germany; ^3^Laboratory for Algae Biotechnology & Innovation, College of Engineering, Peking University, Beijing, China; ^4^Enzyme Technology and Protein Bioinformatics Laboratory, School of Biotechnology, Institute of Science, Banaras Hindu University, Varanasi, India; ^5^State Key Laboratory of Biocatalysis and Enzyme Engineering, School of Life Sciences, Hubei University, Wuhan, China

**Keywords:** microalgae, chassis cell, synthetic biology, metabolic engineering, photosynthetic production

## Introduction

Microalgae play an important role in the oxidation and carbon-reduction process of the Earth's atmosphere, and they currently remain as the essential source of primary productivity to maintain the stability and development of the biosphere. In the context of increasing global energy and environmental crises, microalgae are also valued as highly promising microbial photosynthetic platforms. Using microalgae for the one-step conversion of CO_2_ and solar energy to biofuels, bio-based chemicals and biomedical products is an attractive new technological paradigm for achieving carbon-neutral sustainable development. Traditionally, the development of microalgal biotechnology relied on the mining and utilization of natural algal species resources, while the rapid development of synthetic biology and metabolic engineering technologies is gradually changing the paradigm of this field. By modifying the natural pathways and introducing heterologous modules, the microalgal metabolism network can be remodeled to relocate the organic carbon flow fixed through Calvin cycle to artificial metabolic pathways for the synthesis of desired products. Meanwhile, by combination of the tailored metabolic and physiological functions of microalgal cells with material- and electrochemical-devices, the application scenario of microalgal biotechnology have been further expanded to the areas of biomedicine and biotherapy, biophotovoltaics and biofuel cells, and aerospace.

However, from the perspective of the overall socio-economic and industrial development level, the achievements mentioned above are generally still the “art products” from “studios” or “small workshops” guided by empirical design. In the R & D processes, a majority of microalgal research and engineering manipulations can hardly be performed in the high-throughout, automated bio-foundry platforms. Regarding the outputs, the efficacy and robustness of microalgal cell factories and biocatalytic systems are usually difficult to compete with the classical heterotrophic systems, and even not yet economically feasible. To accelerate the development and industrialization of advanced microalgal biotechnologies, which satisfies the requirements of both sustainability and economic-feasibility, breakthroughs in approaches and platforms are required in the existing microalgal research and engineering system, and the development of universal chassis cells will play a key role. Toward the development and application of the new generation microalgal chassis cells, our understandings on the function and particularly regulation of microalgal genetics, physiology, and metabolism are yet to be expanded, the toolboxes for microalgal genome engineering need to be updated and enriched, and efficiency and robustness of current microalgal cell factories should be significantly improved. The publications in this Research Topic focus on addressing issues for *Engineering microalgal chassis cells*.

Currently, microalgae used for synthetic biology and metabolic engineering research are mostly unicellular species, mainly because of the morphology characteristics facilitating convenient genetic modifications and physiological and biochemical assays. However, unicellular microalgal strains would face difficulties in harvesting, poor resistance to adversity, and predation by protozoa, when being cultivated in outdoor and industrial conditions. In recent years, filamentous microalgae have gained attention due to their ideal industrial properties, and the development of filamentous microalgae chassis cells is important for enriching the microalgae synthetic biotechnology system. Bozan et al. sequenced a filamentous diazotrophic cyanobacterium *Tolypothrix* sp. PCC 7712, which is suited for cultivation with the capillary biofilm reactor (CBR) to reach high biomass production rates. Surprisingly, although PCC 7712 was identified to possess top performance in CBRs cultivation, its genome sequence has a high similarity to that of another species *Tolypothrix* sp. PCC 7601 (*Fremyella diplosiphon*), and the detailed comparative genomic analysis revealed that the physiological differences between the two strains might be resulted from the deviations on gene composition and arrangement. Understandings on genome sequence and characteristics of PCC 7712 would also facilitate genomic modification and metabolic engineering of this strain in future, leading to robust photosynthetic biomanufacturing processes suitable for industrial applications.

Compared to the classical heterotrophic microorganisms, tools for understanding, engineering and harnessing microalgae, are still limited. Developing a suited biotechnology toolbox would be a prerequisite for engineering and applying microalgal chassis cells. Transposon insertion mutagenesis is an important approach used to explore gene functions in microalgae, and Hu et al. reviewed the application of transposon insertion site sequencing method aiming to provide theoretical and technical support when using this strategy. The availability of appropriate plasmid vectors sets is of great significance for introducing and expressing heterologous proteins in microalgae. Sakamaki et al. reported the characterization of a cyanobacterial Rep protein (a replication initiating factor) in the model cyanobacterium *Synechocystis* sp. PCC 6803, which exhibits high autonomous replication activity in multiple cyanobacteria, and established a robust expression vector with this protein. Kaltenbrunner et al. discovered that the copy number of pSYSA defense plasmid in *Synechocystis* sp. PCC 6803 is positively related with the expression level of endoribonuclease E, and identified another protein determining the stability of this plasmid. The information and devices given in the above research would benefit the development of novel shuttle vectors for genetic engineering of cyanobacteria. Besides the “hardware” facilitating direct genetic manipulations, genome metabolic model as a “software” is playing an increasingly important role in microbial synthetic biology and metabolic engineering research. Inwongwan et al. summarized the progress and challenge on developing *Euglena gracilis* metabolic network model, and prospected the future application of this approach to guide precise metabolic engineering. Santos-Merino, Gargantilla-Becerra et al. reported the development of an updated genome-scale model of a freshwater cyanobacterium *Synechococcus elongatus* PCC 7942, and evaluated the potentials of this chassis strain for production of α-linolenic acid. Such a model could also be expected to benefit the development of photosynthetic cell factories of many other metabolites.

Microalgal cells would be challenged by multiple environmental stress factors, and thus it is important to improve the cellular robustness and fitness of the microalgal chassis cells, aiming to achieve rapid and stable carbon fixation, cell growth, and photosynthetic production in diverse conditions. Cantrell et al. reported that disruption of glycogen cycle or sucrose cycle in PCC 6803 led to redirected cellular energy for faster growth under high light conditions, which would also relieve the cell growth retardation caused by the high light stress in outdoor conditions. Dong et al. introduced a heterologous pathway for ectoine synthesis in PCC 7942 and improved salt tolerance of this strain, which might improve the performance of PCC 7942 cell factories when using sea-water for cultivation. Besides harmful environmental factors, the accumulation of products or intermediate metabolites would also cause inhibition on cell growth and metabolism of microalgal cells. Zhang H. et al. reported that expression of tardigrade disordered proteins (TDPs) from water bears could regulate the tolerance of PCC 6803 toward biofuels and metal ions. Through modification of the native metabolism network or introduction of heterologous modules, the physiological adaptation space of microalgal chassis cells and cell factories were effectively expanded, and more robust photosynthetic biosynthesis process could be expected.

The remaining publications in this topic focused on engineering microalgae for photosynthetic production of diverse natural or non-natural metabolites. Zhu et al. summarized the trends and progress on developing microalgal cell factories for lipids production and accumulation through metabolic and process optimizations. In another review article, Santo-Merino, Yun et al. focused on photosynthetic production of sucrose with cyanobacteria; the authors gave a comprehensive review of the current understandings about sucrose metabolism network and regulatory mechanism, summarized the genetic manipulation efforts to optimize sucrose titers, and prospected the trends in developing artificial consortia based on cyanobacterial sucrose secretion. Aiming to guide the optimization the carotenoids production microalgae, Zhang Z. et al. deciphered the role of cryptochromes as photoreceptors to mediate the blue light induced biosynthesis of carotenoids in *Phaeodactylum tricornutum*, and Jiang et al. reported that the overexpression of a plastid lipid-associated protein (PAP) led to optimization of xanthophyll synthesis and accumulation in the same strain. In another study, Opel et al. reported the efforts to develop a synthetic hydrogen sensor in cyanobacteria by introducing the *Cupriavidus nicators* sourced oxygen-tolerant regulatory hydrogenase, which showed distinct H_2_ oxidation activity in PCC 6803. Although the complete H_2_ sensing cascade is yet to be established in cyanobacteria, a functional circuit was successfully constructed in *Escherichia coli* utilizing the associated two-component system. When the functional H_2_ biosensor system was finally constructed and implemented in cyanobacteria in future, it would undoubtedly be an important contribution to the development and optimization of hydrogen-producing cell factories.

Overall, the publications in this topic presented the efforts, progresses, and challenges to engineer a new generation of microalgae chassis cells, which would lay a foundation to develop more efficient and robust microalgal biotechnologies and pave the way for more sustainable manufacturing routes of energy and materials.

## Author contributions

XL drafted this editorial and all authors are participated in the revision. All authors approved the final version.

